# Very Low Frequency of PAD in People with CHD in Six Middle Eastern Countries

**DOI:** 10.1155/2012/409057

**Published:** 2012-03-27

**Authors:** Hasan Kutsi Kabul, Ilker Tasci

**Affiliations:** ^1^Department of Cardiology, Gulhane School of Medicine, 06018 Ankara, Turkey; ^2^Department of Internal Medicine and Geriatrics, Gulhane School of Medicine, 06018 Ankara, Turkey

We read with interest the article by Al-Thani et al. [1] reporting a very low frequency (2.6%) of peripheral arterial disease (PAD) in people with acute coronary syndrome in a multicenter study conducted in six Middle Eastern countries. A recent multicenter study in Turkey showed 20% prevalence of PAD in people with established cardiovascular disease or who were at risk for atherosclerotic diseases [2].

Diabetes that was present in 70% of the participants in their study is actually a major cause of arterial stiffness or calcification, which frequently results in misclassification of diabetics in ankle brachial index (ABI) testing by causing paradoxically high values [3]. In this context, it would be utmost helpful if the authors could provide some data related to the prevalence of a high ABI (>1.4) in their study population, so that the readers could recognize whether a high ABI potentially influenced the frequency of a low ABI.

Another point that should be noted is the method of assessment of PAD by ABI testing. In recent years, many societies published detailed consensus guidelines on the definition and management of PAD [4–6]. Collectively, the ABI cut-off value for the diagnosis of PAD is currently accepted as ≤0.9. Although ABI of <0.8 can be found in several surveys as the diagnostic threshold, adherence to guidelines and their updates would be a superior evidence-based approach. In such case, it could be speculated that the prevalence of PAD would be recorded some higher in the study by Al-Thani et al.

A final concern is the need for a correct calculation of ABI. As explained clearly in the TASC II guidelines, [4] higher readings of dorsalis pedis *or* tibialis posterior arteries should be used as the numerator in the index formula. However, Al-Thani et al. used the *average *(i.e., mean of dorsalis pedis plus tibialis posterior records) systolic blood pressure readings in each ankle as the numerator in order to calculate the ABI ratio. Compared to guideline recommendations, this method obviously results in use of lower values for any ankle especially when one of the readings is lower than the other, causing an increase in the frequency of a low ABI. Opposite to our concern regarding the ABI cut-off in the previous section, such a calculation could further reduce the reported prevalence of PAD in this multicenter large scale survey. However, through a scientific view, this does not eliminate the potential limitations. We guess that the readers would appreciate if the authors could comment on these issues.
